# Diagnostic challenges in synovial sarcoma: a case report

**DOI:** 10.1186/s13256-026-06267-6

**Published:** 2026-06-21

**Authors:** Johan Diaz, Christy Sanchez, Vielka Fernandez, Monica Recine, Natassja Gangeri

**Affiliations:** 1 Department of Internal Medicine, Mount Sinai Medical Center of Florida Inc, 4300 Alton Rd, Miami Beach, FL 33140 USA; 2 Department of Pathology, Mount Sinai Medical Center of Florida Inc, Miami Beach, FL USA

**Keywords:** Synovial sarcoma, Metastasis, Lungs, Leg, Case report

## Abstract

**Background:**

Synovial sarcoma (SS) is a rare, aggressive malignant neoplasm with a high rate of metastasis despite its indolent nature. A distinct SS18::SSX fusion oncogene characterizes SS. The monophasic subtype is the most common and consists of spindle cells. Due to its rarity and subtle clinical presentation, SS can be challenging to identify, leading to misdiagnosis or delays in treatment. The present case highlights the importance of awareness and vigilance among healthcare providers when encountering slow-growing, seemingly benign soft-tissue tumors.

**Case presentation:**

A 43-year-old Haitian man experiencing homelessness with a history of psychiatric disorder, tuberculosis, and substance use presented to the emergency department with right leg pain and intermittent chest pain. Physical examination revealed a large, tender mass on his calf, initially considered benign in past encounters. However, further imaging (CT and MRI scans) showed a large 14.3 cm, lobulated, heterogeneous soleus intramuscular mass with multiple peripheral and internal calcifications suggestive of soft-tissue sarcoma. In addition, a CT chest revealed multifocal pulmonary nodules highly suspicious for metastatic disease. Histologic examination of lung biopsies showed cells positive for vimentin, BCL-2, and TLE-1, consistent with monophasic SS; molecular confirmation of the SS18::SSX fusion was not obtained. Systemic AIM chemotherapy (doxorubicin, ifosfamide, and mesna) was planned. However, the patient declined further care and was lost to follow-up. He was subsequently reported to have died from pulmonary complications of metastatic disease approximately 6 months later.

**Conclusions:**

This case highlights the complexity of diagnosing SS, given its subtle clinical findings, slow growth, and atypical presentation. Multidisciplinary care is essential for optimizing diagnosis and treatment outcomes. Prompt recognition and comprehensive care can improve patients’ prognosis.

**Supplementary Information:**

The online version contains supplementary material available at https://doi.org/10.1186/s13256-026-06267-6.

## Background

Synovial sarcoma (SS) is a rare, malignant soft-tissue neoplasm, representing 5–10% of all soft-tissue sarcomas, and is among the most common lower extremity malignancies in young individuals [1–3]. Approximately 80–95% of SS occur primarily in the extremities, especially in the lower limbs near the knee, although primary lesions can also arise in the thorax, head, or neck [2]. SS neoplasms are aggressive, showing high local recurrence rates and a tendency for pulmonary and osseous metastasis [2]. Five-year survival varies from 24 to 71%, influenced by patient age, tumor size, anatomic location, and histology [2].

Although their name suggests otherwise, SS do not arise from synovial cells and are rarely located within joint spaces [2, 3]. SS tumors are mesenchymal spindle cell neoplasms showing various levels of epithelial differentiation [2, 4]. About 95% of these tumors are characterized by a distinct SS18::SSX fusion oncogene formed through a chromosomal translocation [1, 2]. SS is commonly categorized into monophasic, biphasic, and poorly differentiated [2]. The most common is the monophasic subtype, consisting primarily of spindle cells [2]. The biphasic subtype contains spindle cells and epithelial cells, while the poorly differentiated subtype consists of cells analogous to small round blue cell tumors [2]. The SS18 protein is an integral subunit of the mammalian SWI/SNF (BAF) ATP-dependent chromatin-remodeling complex [5, 6]. When fused to the C-terminus of SSX, the resulting SS18::SSX oncoprotein is incorporated into BAF complexes in place of wild-type SS18 and evicts the tumor-suppressor subunit BAF47 [5, 6]. The aberrant complex is then retargeted across the genome, activating a synovial sarcoma-specific transcriptional program that drives tumorigenesis [6]. According to the World Health Organization Classification of Soft Tissue and Bone Tumors, synovial sarcoma is characterized by recurrent SS18::SSX fusions [7]. Molecular detection of this fusion provides strong diagnostic confirmation, particularly in monophasic or poorly differentiated cases [4].

Approximately one to two people per million are diagnosed with SS annually in the United States [1]. SS rarity and often subtle clinical presentation contribute to the substantial challenges in diagnosing and promptly treating this disease [1, 2]. The present case report documents a case of adult SS with lung metastasis whose initial presentation and indolent progression delayed his diagnosis. This case emphasizes the importance of awareness and vigilance among healthcare providers when encountering slow-growing, seemingly benign soft-tissue tumors.

## Case presentation

A 43-year-old Haitian man experiencing homelessness with a medical history of psychiatric disorder on medications, substance use disorder, and tuberculosis treated 3 years ago, presented on May 29th, 2024, to the emergency department (ED) with severe right leg pain radiating from the calf to the hip and intermittent chest pain. The leg pain, constant and dull (8/10 in severity), had been progressing for over 2 years. The patient identified a focal swelling approximately 11–13 cm in size on his right calf as the source. He reported a distant trauma history involving a pedestrian car accident resulting in a distal right leg hematoma without fractures in 2014. Chest pain began 2–3 weeks prior without specific triggers or relief measures. However, he reported a recent hospital admission after experiencing fatigue, shortness of breath, and hemoptysis, which resolved spontaneously at another facility. The patient reported no known family history of malignancy; however, this history was difficult to verify given limited health literacy and restricted access to family medical records. His psychosocial history was notable for prolonged housing instability and limited prior engagement with the healthcare system.

At presentation, vital signs were normal, with adequate oxygenation on room air. Examination revealed a focal swelling at the proximal posterior right calf, with intact overlying skin and regular pulses throughout the lower extremity. There were no thrills or bruits with a normal joint range of motion, but tenderness on right knee flexion was caused by mass compression. Lungs and cardiovascular examination were normal. Labs indicated microcytic anemia from iron deficiency. Infectious evaluations showed negative blood cultures, HIV, and hepatitis tests. QuantiFERON was positive, but AFB cultures and FTA–ABS were negative. The leg pain was attributed to mechanical compression by the enlarging mass rather than to a neuropathic process, as the examination demonstrated no sensory loss, paresthesia, or motor weakness, and distal pulses and the joint range of motion were preserved.

**Fig. 1:**
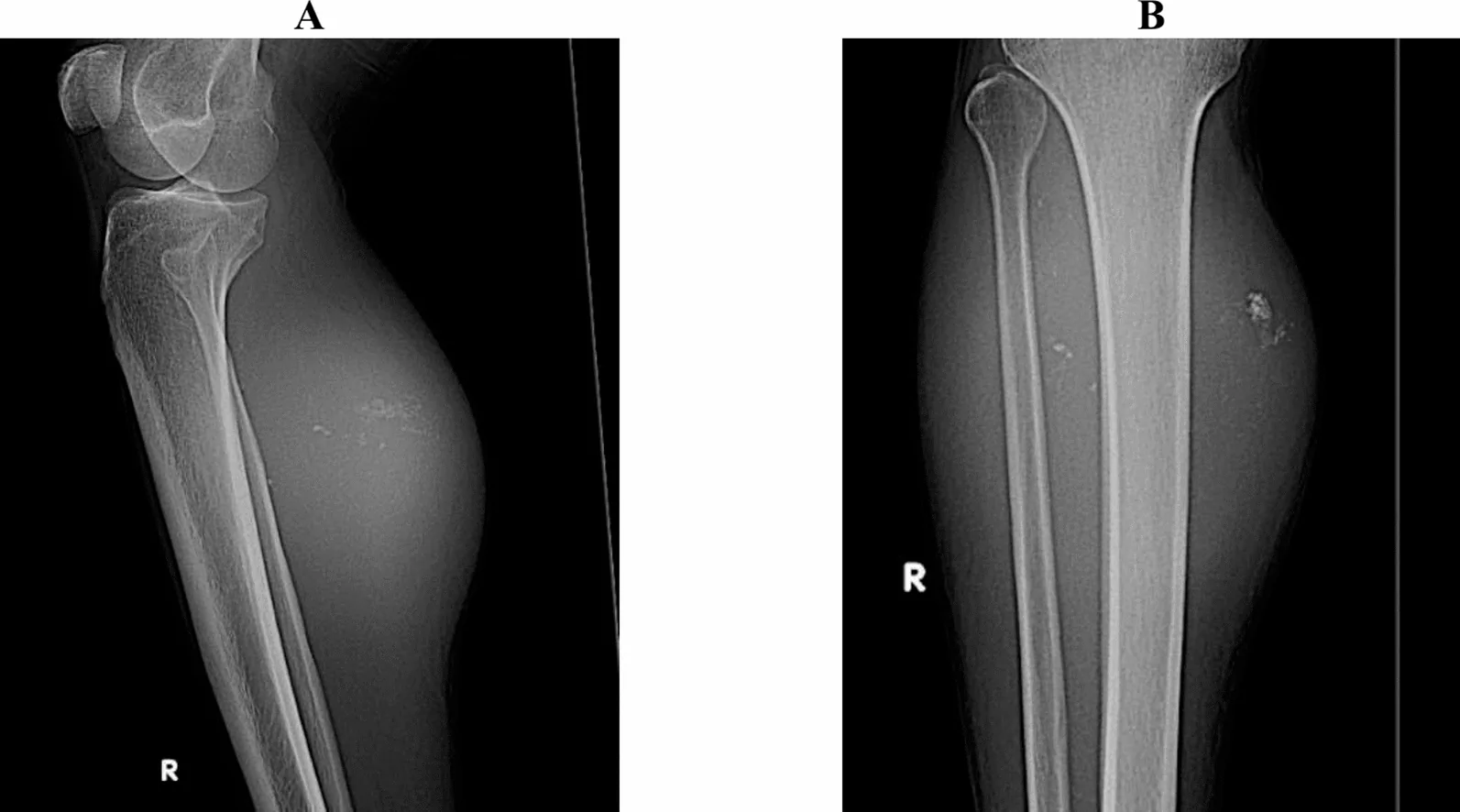
Patient’s right-lower extremity X-ray results at presentation on May 14th, 2024. **A,** **B** Ill-defined lesion with scattered calcifications within the calf

**Fig. 2:**
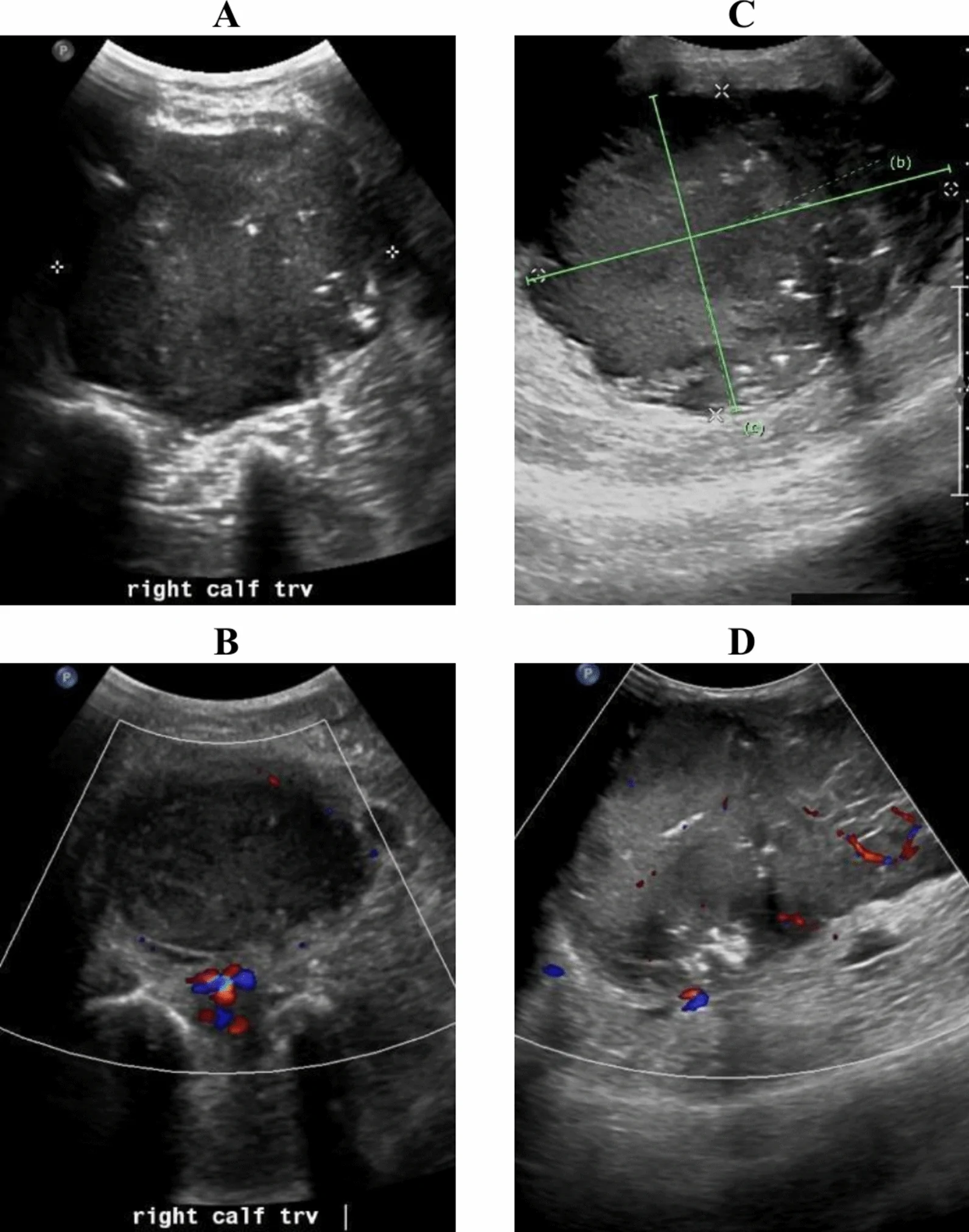
Patient’s ultrasound results in May 2024. **A,** **B** Well-defined 5.7 × 8.0 × 11.8 cm right medial calf complex structure with calcifications and vascularity on May 14th. **C,** **D** Same structure minimally increased in size, 8.0 × 8.6 × 11.5 cm on May 29th

**Fig. 3:**
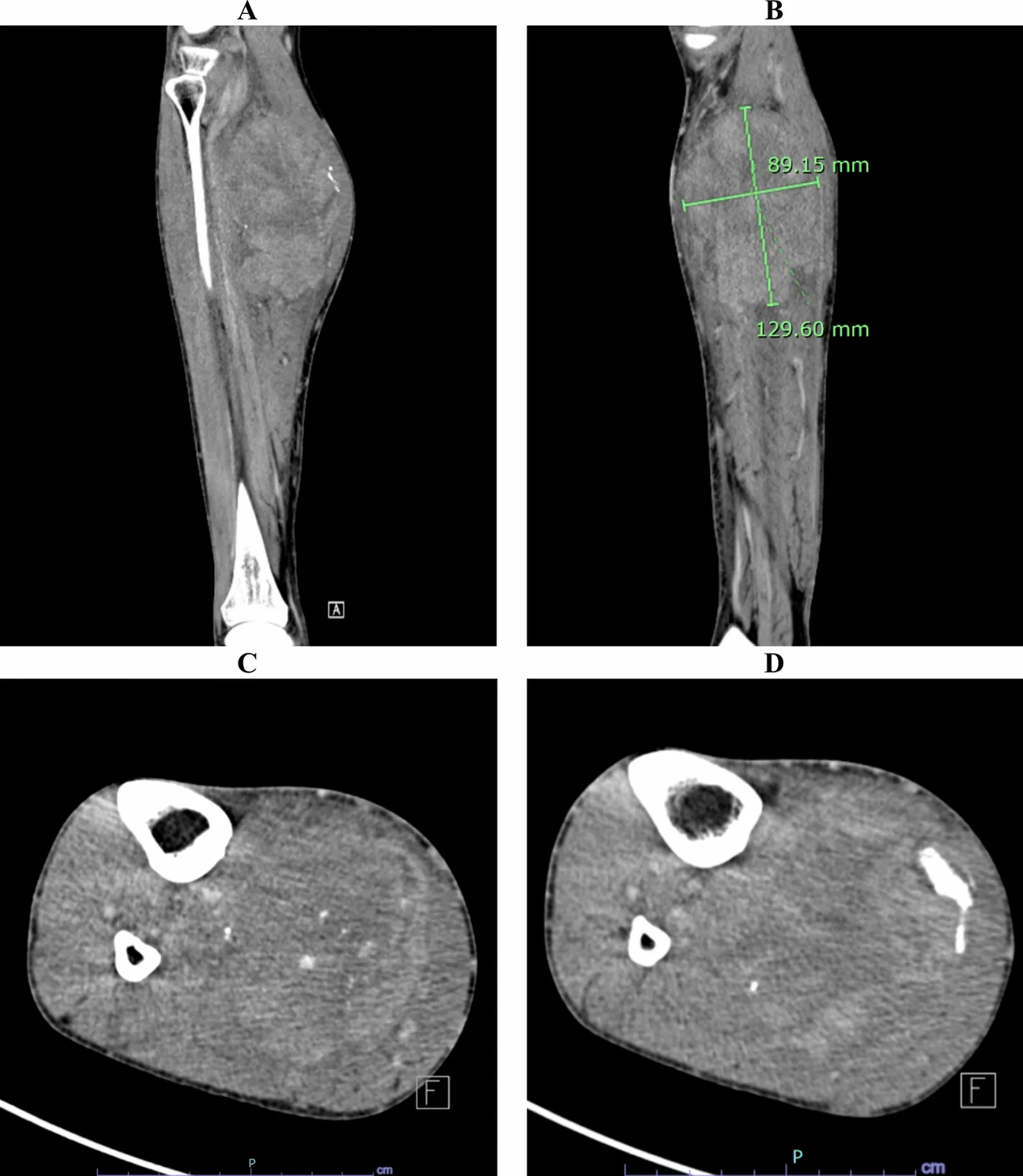
CT of the right tibia and fibula obtained on May 14th, 2024, showing an 8.9 × 8.7 × 13.0 cm lobulated and heterogeneous soleus intramuscular mass with multiple peripheral and internal calcifications in coronal (**A)**, sagittal (**B)**, and two axial views (**C)** and (**D)**

Two weeks prior, the patient presented to the ED on May 14th with similar complaints. An X-ray revealed an ill-defined calf lesion with calcifications (Fig. 1). Duplex ultrasound excluded deep vein thrombosis, showing a 5.7 × 8.0 × 11.8 cm vascular medial calf lesion, concerning for a hemangioma or venous malformation (Fig. 2A, B). CT demonstrated a 13.0 cm lobulated, heterogeneous soleus intramuscular mass with calcifications (Fig. 3). Following adequate symptomatic treatment, arrangements were made for the patient to follow up as an outpatient with his primary doctor and surgery team, but he missed both appointments. The patient’s clinical course over the preceding decade is summarized in Table 1.

**Table 1:** Timeline of the patient’s clinical course (2014–2024)

Date	Clinical events
2014	Pedestrian motor-vehicle accident; right distal-leg hematoma, no fracture
October 2015	ED visit. US (right calf): 2.4 × 1.4 × 2.9 cm, calcified and vascular complex structure; read as complex cyst/seroma/hematoma. Outpatient follow-up missed
June 2016	ED visit. US (right calf): 4.0 × 1.3 × 3.1 cm; same benign interpretation. Outpatient follow-up missed
May 14th, 2024	ED visit. Radiograph: calcified calf lesion. Duplex US (right calf): 5.7 × 8.0 × 11.8 cm mass; DVT excluded. CT: 8.9 × 8.7 × 13.0 cm soleus mass with calcifications. Outpatient primary-care and surgery follow-up missed
May 29th, 2024	ED visit and hospital admission. US (right calf): 8.0 × 8.6 × 11.5 cm complex structure. CXR/CT angiography: innumerable bilateral pulmonary nodules; right-lower-lobe mass. MRI: 8.8 × 8.6 × 14.3 cm soleus mass. CT-guided lung biopsy: spindle cells, vimentin + /BCL-2 + /TLE-1 + , consistent with monophasic synovial sarcoma
June 2024	AIM chemotherapy planned. Patient left against medical advice
November 2024	Reported deceased under palliative care at an outside facility

**Fig. 4:**
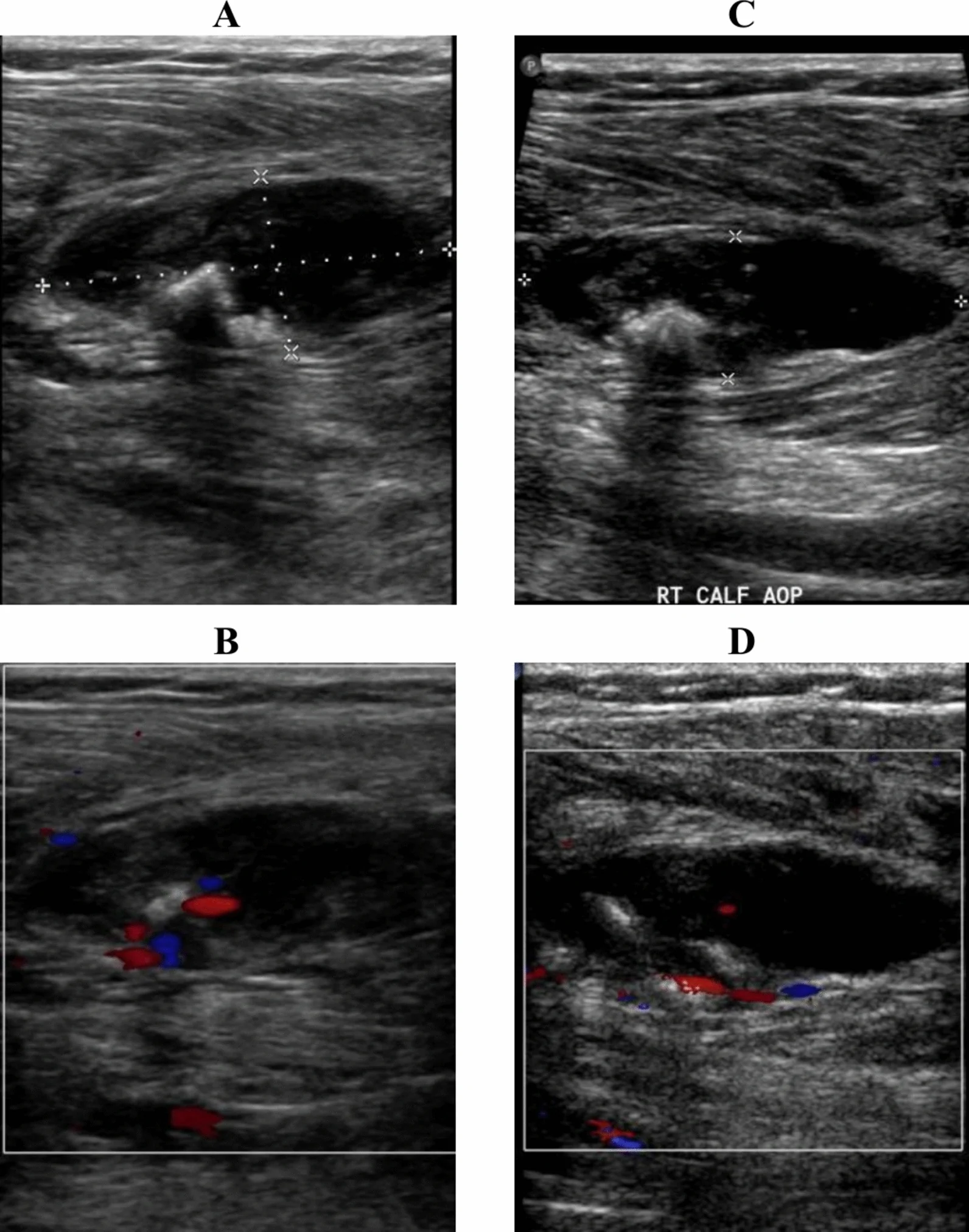
Patient’s prior ultrasound results. **A,** **B** Right-sided medial calf structure measuring 2.4 × 1.4 × 2.9 cm with calcifications and minimal vascularity in October 2015. **C,** **D** Same structure now measuring 4.0 × 1.3 × 3.1 cm in June 2016

A comprehensive chart review revealed two prior ED encounters in October 2015 and June 2016, where the patient presented with complaints of right-lower extremity pain. In October 2015, an ultrasound showed a right-sided medial calf structure measuring 2.4 × 1.4 × 2.9 cm with calcifications and vascularity (Fig. 4A, B). Then, in June of 2016, ultrasonography showed a right medial calf subcutaneous complex lesion with calcifications and minimal vascularity measuring 4.0 × 1.3 × 3.1 cm, increased in size compared to his prior ultrasound (Fig. 4C, D). At the time, these findings were believed to represent a complex popliteal cyst versus seroma or hematoma. Similarly, the patient was discharged with plans for outpatient follow-up during both encounters but missed both appointments.

**Fig. 5:**
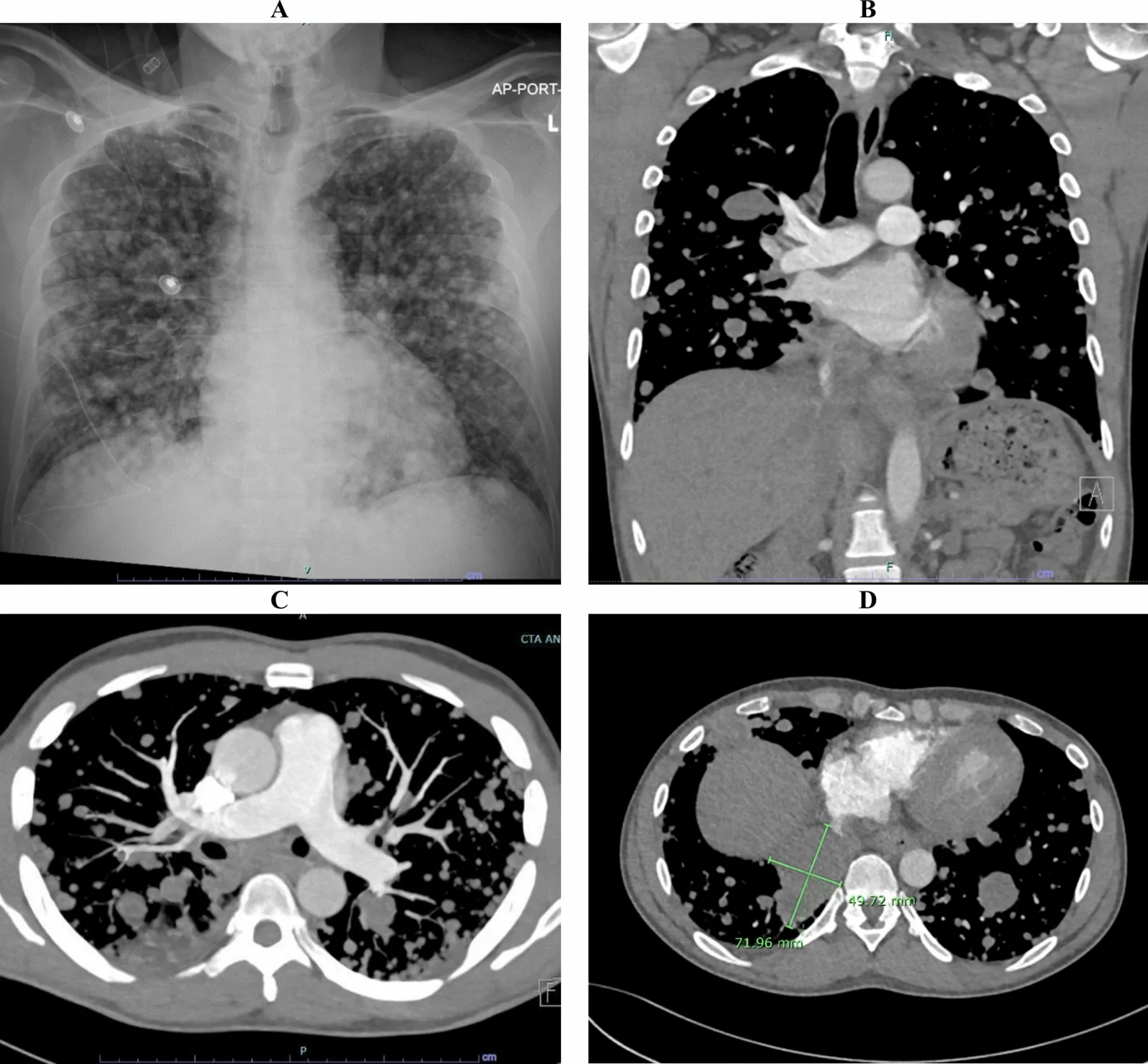
**A** Chest X-ray with innumerable, randomly distributed pulmonary nodules throughout both lungs. **B,** **C** Coronal and axial views of a CTA chest showing innumerable pulmonary nodules scattered throughout the lungs in a random distribution, mildly prominent mediastinal, and bilateral hilar lymph nodes. **D** Axial view showing dominant mass-like opacity at the basilar segments of the right-lower lobe, measuring approximately 5.0 × 7.2 × 6.1 cm

During the present admission, a repeat ultrasound showed growth of the lesion, now measuring 8.0 × 8.6 × 11.5 cm (Fig. 2C, D). A chest X-ray revealed numerous bilateral pulmonary nodules suspicious for metastatic disease (Fig. 5A). CT angiography showed no pulmonary embolism but confirmed multiple randomly distributed pulmonary nodules, a 7.2 cm dominant mass-like opacity in the right-lower lobe, possible left atrial invasion, and bilateral enlarged lymph nodes (Fig. 5B–D). Further CT scans ruled out abdominal, pelvic, and brain metastases, while echocardiography excluded cardiac invasion.

**Fig. 6:**
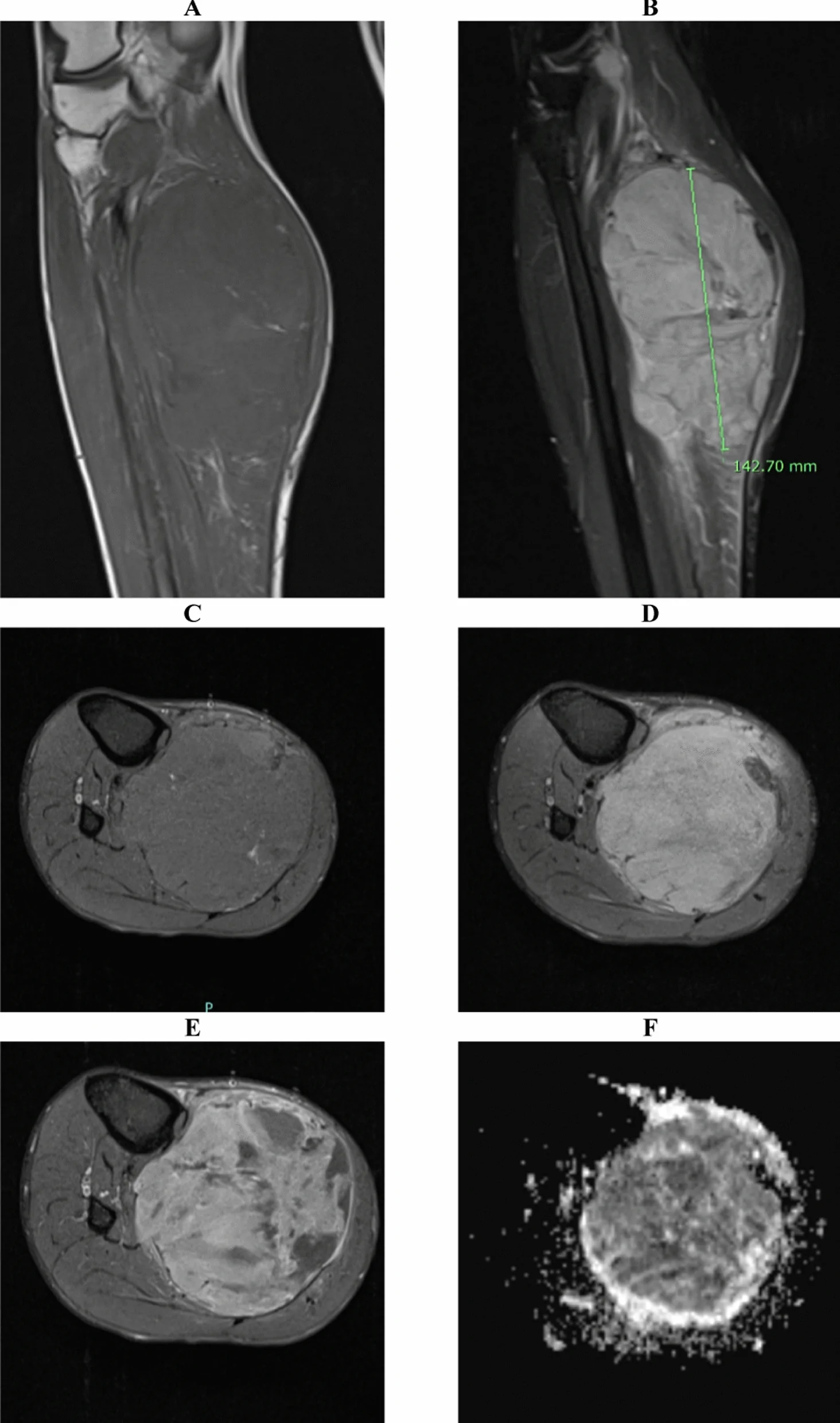
**A,** **B** T1-weighted and STIR coronal views of a large multilobulated soft-tissue mass measuring 8.8 × 8.6 × 14.3 cm. **C** Pre-contrast T1-weighted axial view, **D** PD axial view, **E** post-contrast T1-weighted axial view, and **F** ADC axial view of the heterogeneous mass

**Fig. 7:**
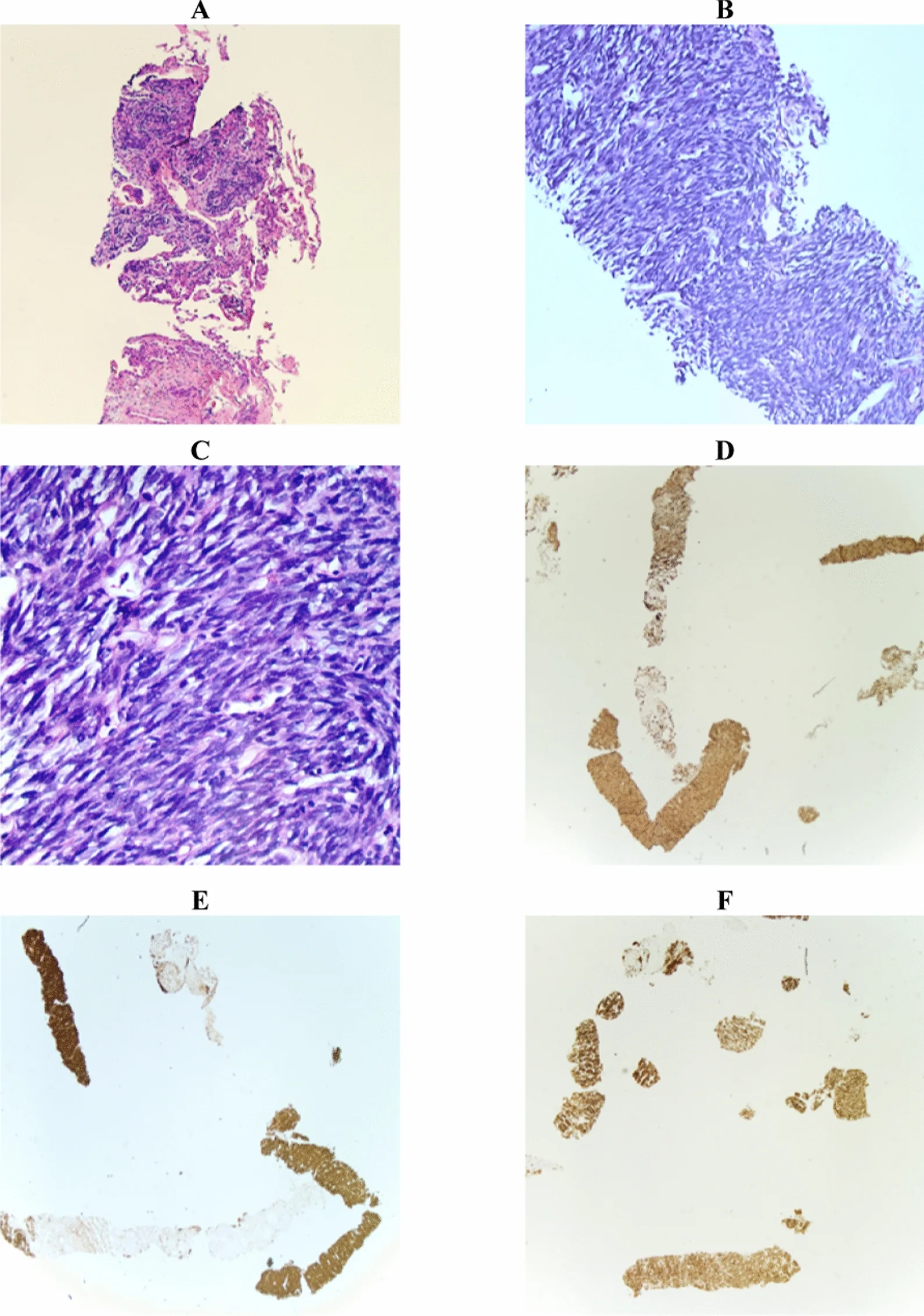
**A**, **B,** **C** Lung parenchyma with an undifferentiated proliferation of spindle cells arranged in fascicles with little intervening stroma (H&E, 10x, 20x, and 40 × objective, respectively). The tumor cells are positive for **D** Vimentin, **E** BCL-2, and **F** TLE-1, supporting the diagnosis of synovial sarcoma, monophasic type

MRI of the right leg detailed a multilobulated 14.3 cm medial soleus mass with enhancement, necrosis, calcifications, and hemorrhage (Fig. 6). A femoral enchondroma and tibial intraosseous cyst were incidentally noted. Next, CT-guided lung biopsies revealed spindle cell proliferation with positive immunostaining for Vimentin, BCL-2, and strong diffuse TLE-1, consistent with monophasic synovial sarcoma (Fig. 7A–F).

Following the diagnosis, the disease was staged clinically. Although a formal Eastern Cooperative Oncology Group performance status was not documented and pathological grading of a resection specimen was not feasible, the combination of a large primary soft-tissue tumor with disseminated bilateral pulmonary metastases was consistent with American Joint Committee on Cancer (8th edition) Stage IV disease. The oncology team recommended systemic therapy with the institution’s standard AIM regimen (doxorubicin 75 mg/m^2^ and ifosfamide 10 g/m^2^ per cycle, with mesna uroprotection, administered every 21 days). Whole-body PET-CT for staging, baseline cardiac assessment, surgical- and radiation-oncology consultation, and central venous access placement were planned but could not be completed. Before treatment began, the patient declined further care and elected to leave against medical advice. Despite multidisciplinary efforts to provide support and to connect him with social and financial resources, he did not return and was lost to follow-up. This outcome was best understood as a consequence of socioeconomic precarity and structural barriers to care rather than simple nonadherence. Subsequent investigation revealed that the patient had been admitted to palliative care at an outside facility and was reported deceased in November 2024, approximately 6 months after diagnosis, from pulmonary complications of his metastatic disease.

## Discussion

Synovial sarcoma is a rare and aggressive malignancy affecting connective tissues, posing significant diagnostic challenges due to nonspecific early symptoms and imaging findings. It predominantly impacts young adults, peaking in incidence during the third and fourth decades of life [1, 2]. The presented case illustrates how subtle initial symptoms contribute to delayed and frequent misdiagnoses. Only about half of SS patients have clinical findings typical of soft-tissue sarcoma, and the mean duration of symptoms before diagnosis has been reported as 98 weeks [8]. Adults also experience frequent misdiagnosis, influenced by tumor size and location [8].

Small SS lesions, particularly in upper limbs, may resemble neurogenic tumors on imaging, while larger lesions (> 5 cm), such as in our patient, commonly appear cystic due to heterogeneous composition [1, 2, 8]. Biopsy accuracy varies significantly, with 9 to 17% misdiagnosis rates, rising to 53.7% when considering cytology alone [8]. Early identification is crucial; delayed diagnoses compromise patient outcomes, as tumors typically present after 2–4 years of gradual growth [2]. In our patient’s initial assessments (2015–2016), ultrasonography revealed a subcutaneous calf lesion initially considered a hematoma or cyst, complicating timely diagnosis (Fig. 4).

Socioeconomic barriers, including the patient’s experience of homelessness, and anchoring bias from a prior trauma history, likely contributed to further delaying diagnosis and the loss of follow-up. In addition, the tumor’s slow progression often falsely suggests benignity [2]. The main metastatic sites are the lungs, lymph nodes, and bone, as metastatic disease significantly worsens survival [1]. Our patient presented with disseminated pulmonary metastases and thoracic lymphadenopathy (Fig. 5). Metastasis drastically reduces survival, emphasizing the importance of vigilant surveillance [3, 9].

Several molecular mechanisms may help explain the size and aggressiveness of the present tumor. Beyond its direct effects on chromatin, the SS18::SSX fusion aberrantly activates canonical Wnt/β-catenin signaling; nuclear β-catenin localization has been reported in 41% of primary and 70% of metastatic synovial sarcomas, supporting a role for this pathway in tumor progression [10]. Sustained growth and metastatic spread also depend on angiogenesis. SS express vascular endothelial growth factor (VEGF) and platelet-derived growth factor receptors [11]. Here, the heterogeneous, hypervascular appearance of our patient’s 14.3 cm mass could be consistent with this angiogenic phenotype. These pathways are of therapeutic interest, as tyrosine kinase inhibitors such as pazopanib are approved for previously treated non-adipocytic soft-tissue sarcoma, including synovial sarcoma [11, 12].

Radiographically, SS typically exhibits calcifications in up to 30% of cases, which, when present, are often fine, stippled, and eccentric or peripheral in distribution (Fig. 1) [2, 11]. Ultrasound findings differ by tumor size: smaller tumors (< 5 cm) appear homogeneous, while larger ones (> 5 cm) display heterogeneous echogenicity with cystic or necrotic areas, hemorrhage, and calcifications (Fig. 4) [2]. In the present case, the coarse peripheral and internal calcifications may represent dystrophic calcification within areas of necrosis and prior hemorrhage, rather than active osteoid production. Our patient’s ultrasounds exhibited progressive mass enlargement with heterogeneous echogenicity, calcifications, and vascularity, suggesting a complex lesion necessitating further imaging examination; however, this was not pursued initially due to loss of follow-up, likely due to socioeconomic barriers.

Computed tomography can better characterize SS by identifying calcifications, heterogeneous enhancement, and regions indicative of necrosis or bleeding [2, 11]. In our case, CT imaging revealed a large intramuscular soleus mass with peripheral and internal calcifications, raising the possibility of SS (Fig. 3). MRI remains the optimal imaging tool for soft-tissue sarcomas, effectively delineating tumor morphology, with typical SS features, including multilobulated, heterogeneous masses exhibiting distinct T2-weighted hyperintensity and specific signs, such as the “triple sign” or “bowl of grapes” appearance [2, 11].

The “bowl of grapes” refers to a multicystic mass with varying fluid intensities and interspersed hypointense septa [2, 11]. Fluid–fluid levels may result from sedimented blood products in a gravity-dependent plane but are not exclusive to synovial sarcoma [2, 11]. The ‘triple sign” is characterized by necrotic, cystic, or hemorrhagic hyperintensity mixed with isointense cellular tissue and hypointense calcified or fibrotic regions; however, it lacks specificity and can also be seen in other soft-tissue sarcomas [2, 8, 11]. Our patient’s MRI showed these features alongside restricted diffusion, suggesting malignancy (Fig. 6).

Histologically, monophasic SS, the most common subtype, comprises spindle cells with minimal cytoplasm and demonstrates distinct immunohistochemical markers, including BCL-2 and TLE-1 positivity, which were confirmed in our patient’s biopsy (Fig. 7) [8, 13]. Although TLE-1 is a sensitive immunohistochemical marker for synovial sarcoma, its specificity is only moderate [13]. Consequently, definitive diagnosis increasingly rests on demonstrating the SS18::SSX fusion by fluorescence in situ hybridization, reverse-transcription PCR, next-generation sequencing, or on fusion-specific immunostain [1, 8, 11]. However, molecular confirmation of the SS18::SSX fusion could not be obtained in this case due to time constrains.

Treatment typically involves surgical resection, radiotherapy, and chemotherapy, particularly for advanced metastatic disease [1, 2]. Anthracycline-based chemotherapy with or without ifosfamide remains standard, achieving higher response rates when combined, though without significant survival improvement [1, 12]. The recent METASYN study confirmed a median progression-free survival of 6 months with chemotherapy in metastatic disease [1]. Beyond conventional cytotoxic therapy, afamitresgene autoleucel, a genetically modified autologous T-cell immunotherapy, received accelerated approval from the United States Food and Drug Administration in 2024 for previously treated, unresectable or metastatic synovial sarcoma in patients with eligible HLA-A*02 types and MAGE-A4-positive tumors, becoming the first FDA-approved T-cell receptor gene therapy [14, 15].

The initial plan was to start our patient on systemic therapy with doxorubicin, ifosfamide, and mesna and continue care as an outpatient. However, due to personal motives, the patient left the hospital against medical advice. Before leaving the hospital, the patient consented to publicly releasing his clinical information and images. Multiple attempts were made to continue care but were unsuccessful. Further investigation revealed that the patient was declared deceased at an outside facility under palliative care in November of 2024, approximately 6 months after his diagnosis.

The decision to leave before treatment could reflect a combination of distrust of the medical system and pressing personal and logistical concerns, including the need to secure belongings he had left at a hotel before admission. He did not voice opposition to the diagnosis itself but felt unable to remain in the hospital at that time, and he did not subsequently return despite efforts to reach him. His experience underscores how mistrust and the daily exigencies of housing and economic insecurity can hinder engagement with potentially life-prolonging oncologic care.

### Strengths and limitations

The principal strength of this report is its longitudinal imaging documentation of an unusually indolent, decade-long course of a tumor that is typically aggressive, illustrating how repeated benign-appearing studies can foster diagnostic anchoring. The report also has important limitations. Most notably, because the patient was lost to follow-up at our institution before systemic therapy could begin, no on-treatment response data are available, and the ultimate outcome was ascertained only retrospectively from outside records, which indicated death from pulmonary complications of metastatic disease approximately 6 months after diagnosis. In addition, the diagnosis rested on characteristic morphology and an immunohistochemical profile (vimentin, BCL-2, and TLE-1 positivity); molecular confirmation of the SS18::SSX fusion was not obtained, which we acknowledge as a diagnostic limitation. Finally, elements of the history, including the family history, could not be fully verified given the patient’s circumstances.

## Conclusion

The presented case underscores the diagnostic complexity of synovial sarcoma, emphasizing the subtle clinical findings, slow tumor growth, and atypical presentations that frequently lead to delayed recognition. Effective multidisciplinary care involving radiologists, pathologists, oncologists, and surgeons is crucial for optimizing diagnostic accuracy and treatment outcomes. Increased clinical vigilance and early, comprehensive management can significantly improve the prognosis for patients.

## Supplementary Information


Supplementary Material 1.

## Data Availability

The article contains all relevant data collected or analyzed during this case report, which were deemed essential for understanding and interpreting the results.

## Abbreviations

ADC: Apparent diffusion coefficient
AFB: Acid-fast bacilli
AIM: Adriamycin (doxorubicin), ifosfamide, and mesna
ATP: Adenosine triphosphate
BAF: BRG1/BRM-associated factor
BAF47: BRG1/BRM-associated factor 47
BCL-2: B-cell lymphoma 2
CT: Computed tomography
CTA: Computed tomography angiography
CXR: Chest X-ray
DVT: Deep vein thrombosis
ED: Emergency department
FDA: Food and Drug Administration
FTA–ABS: Fluorescent treponemal antibody absorption
H&E: Hematoxylin and eosin
HIV: Human immunodeficiency virus
HLA-A*02: Human leukocyte antigen-A*02
IRB: Institutional Review Board
MAGE-A4: Melanoma-associated antigen A4
METASYN: METAstatic SYNovial Sarcoma
MRI: Magnetic resonance imaging
PCR: Polymerase chain reaction
PD: Proton density
PET–CT: Positron emission tomography–computed tomography
SS: Synovial sarcoma
SS18: Synovial sarcoma translocation, chromosome 18 gene/protein
SS18::SSX: Fusion oncogene formed by SS18 and an SSX gene
SSX: Synovial sarcoma X breakpoint gene family
STIR: Short tau inversion recovery
SWI/SNF: Switch/sucrose non-fermentable
TLE-1: Transducin-like enhancer of split 1
US: Ultrasound
VEGF: Vascular endothelial growth factor
Wnt: Wingless-related integration site
